# Enzymolysis-Driven Development of a Gut-Targeted *Aronia melanocarpa* Meal Replacement Powder with Glycemic Control and Microbial Homeostasis Benefits

**DOI:** 10.3390/foods14142456

**Published:** 2025-07-12

**Authors:** Yongxing Li, Zhihui Hu, Haiyu Ji, Shuang Yang, Ruihan Guo, Jinfang Zhang, Hongjun He, Bo Xu, Mei Li

**Affiliations:** 1 Center for Mitochondria and Healthy Aging, College of Life Sciences, Yantai University, Yantai 264005, China; liyongxing0826@163.com (Y.L.); huzhihui9968@163.com (Z.H.); haiyu11456@163.com (H.J.); syang979797@163.com (S.Y.); grh201900974@163.com (R.G.); hemiles@163.com (H.H.); 2 School of Food Engineering, Ludong University, Yantai 264025, China; jfzhang1007@163.com

**Keywords:** meal replacement powder, enzymatic hydrolysis, glycemic index, gut microbiota

## Abstract

In this study, the effects of enzymolysis on physicochemical properties, digestive characteristics, and flora regulation of the meal replacement powder (MRP) were investigated on the basis of the previously obtained compound MRP. The results showed that the color, water absorption index, and water solubility index of the MRP were obviously improved after enzymatic hydrolysis. The swelling power (1.43 ± 0.11 g/g, 25 °C) and water-holding capacity (4.66 ± 0.09 g/g) of the MRP (CE_1) were decreased, while the oil holding capacity (2.14 ± 0.13 g/g) was increased. In the microcosmic aspect, the samples treated by enzymolysis had different degree of degradation, the particle size decreased (D50 = 57.71 μm), and the specific surface area (679.2 cm^2^/g) increased. The MRP samples treated by enzymolysis had better antioxidant capacity and cholate adsorption capacity. All MRP samples belong to low glycemic index (GI) foods, and can improve gut microbiota (*Megamonas*, *Bacteroides*, *Rocheella*, *Parasatre*, *Koalabacterium*, and *Prasus*) and promote the production of short chain fatty acids such as acetic acid, propionic acid and butyric acid. Therefore, this study not only further expands the comprehensive utilization of *Aronia melanocarpa*, but also provides a reference for the diversification of low GI related products.

## 1. Introduction

The recently released World Obesity Map 2023 from the World Obesity Alliance presents alarming projections: global overweight and obesity prevalence is predicted to escalate from 2.6 billion affected individuals in 2020 to exceeding 4 billion by 2035, potentially impacting 51% of the global population [1]. As an established independent risk factor for multisystemic chronic diseases, obesity substantially increases vulnerability to metabolic disorders including type 2 diabetes, non-alcoholic steatohepatitis, and cardiovascular complications [2]. While chronic energy surplus remains a principal etiological driver, contemporary research underscores dietary fiber insufficiency as a critical modulator in obesity pathophysiology. Mechanistic studies reveal that high-fiber dietary patterns exert beneficial effects on appetite regulation and weight control through multiple pathways, particularly via enhancing satiety signaling and gut microbiota modulation [3].

Meal replacement powders (MRPs), predominantly composed of plant-based ingredients such as cereals, legumes, and tuber crops, have emerged as scientifically formulated alternatives for weight management, offering optimized nutrient profiles with controlled caloric density [4]. These functional food products are specifically engineered to provide enhanced levels of insoluble and soluble dietary fibers, essential micronutrients, and a balanced mineral matrix containing trace elements [5]. Current innovation in MRP development focuses on three key functional characteristics: glycemic response modulation through low glycemic index (GI) formulations, anti-inflammatory activity via phytonutrient enrichment, and energy density reduction. Seminal clinical investigations by Heymsfield et al. [6] demonstrate that micronutrient-fortified MRPs not only facilitate weight reduction but also ameliorate obesity-related comorbidities, particularly components of metabolic syndrome, thereby positioning them as valuable adjuncts in chronic disease management strategies. Despite these advancements, conventional MRPs face limitations including nutritional imbalance, organoleptic imperfections (particularly textural coarseness), and insufficient gastrointestinal functionality enhancement [7].

Enzymatic hydrolysis plays a key role in addressing these challenges and has become an important strategy for the functionalization of meal replacement powders. Cellulase-assisted bioprocessing enhances phytochemical bioavailability and oxygen radical absorbance capacity via targeted degradation of lignocellulosic matrices, while synergistic α-amylase/glucoamylase hydrolysis achieves controlled depolymerization of starch macromolecules into defined low-molecular-weight dextrins and mono/oligosaccharides [8]. This enzymatic approach offers advantages in nutrient bioavailability enhancement, product stability improvement, and sensory attributes modification while alignment with sustainable manufacturing principles (reduced chemical auxiliaries) [9].

*Aronia melanocarpa* is a valuable species integrating edible, medicinal, landscaping, and ecological protection values, widely utilized in the medicine and food industries. Its fruits are rich in organic acids, polyphenols, sugars, and other compounds [10]. Previous pharmacological studies have revealed that the polyphenolic compounds in *Aronia melanocarpa* fruit exhibit antioxidant, immunomodulatory, hypoglycemic, and hypotensive effects [11]. The berries contain higher concentrations of anthocyanins, protoprotein, phenolic acids and dietary fiber (DF) and have stronger antioxidant properties than common berries such as blueberries and cranberries [12]. Mechanism studies have shown that DF can reduce attenuate postprandial hyperglycemia through viscosity-mediated delayed carbohydrate absorption and short-chain fatty acid-mediated insulin sensitivity improvement [13]. Notably, its distinctive phytochemical composition positions it as an ideal candidate for developing functional MRPs with combined weight management and chronic disease prevention properties [14].

This study aims to develop an enzymatically modified *Aronia melanocarpa*-based MRP with enhanced gastrointestinal functionality and antioxidant capacity. Through systematic optimization of enzymatic processing parameters and formula composition, we seek to address current MRP limitations in palatability and nutrient bioavailability. Our multidisciplinary approach evaluates the product’s physicochemical properties, *in vitro* digestibility, and gut microbiota modulatory effects, while assessing its nutritional composition, bioactivity, and safety profile. This research provides critical insights for developing next-generation functional MRPs targeting weight management, metabolic syndrome amelioration, and gut health optimization, ultimately contributing to personalized nutrition strategies for chronic disease populations.

## 2. Materials and Methods

### 2.1. Experimental Materials

The red adzuki beans and black rice used in this study were purchased in bulk from a local Aeon supermarket and confirmed to be fresh and mold-free. After washing, the raw materials were subjected to steaming (red adzuki beans) and stir-frying (black rice) to cook, followed by drying at 60 °C, milling, and sieving through a 100-mesh sieve. The processed powders were then stored at 4 °C until further use. *Aronia melanocarpa* was provided by Weihai Blue Economy Research Institute Co., Ltd. (Weihai, China). Cellulase (4 × 10^5^ U/g, CAS: 9012-54-8) was purchased from Shanghai Macklin Biochemical Co., Ltd. (Shanghai, China). Trypsin (potency ≥ 250 U/mg, CAS: 9002-07-7) was purchased from Shanghai Aladdin Biochemical Technology Co., Ltd. (Shanghai, China). Pepsin from porcine gastric mucosa (15000 U/g, CAS: 9001-75-6), α-amylase from porcine pancreas (11 U/mg, CAS: 9000-90-2), amyloglucosidase (1 × 10^5^ U/g, CAS: 9032-08-0) were all purchased from Shanghai Yuanye Bio-Technology Co., Ltd. (Shanghai, China). All other materials and reagents were analytical grade.

### 2.2. Preparation of Meal Replacement Powder

In the early stage, low GI meal replacement powder (red beans (52.73%), black rice (39.96%) and *Aronia melanocarpa* (7.31%)) was prepared by sensory evaluation based on low energy, low fat and high dietary fiber. Previous orthogonal experiments identified the compound enzyme addition amount as the most significant influencing factor, with optimal hydrolysis conditions determined as follows: compound enzyme ratio of 1:3, a hydrolysis time of 40 min, and a temperature of 50 °C (Table S1). Based on these results, meal replacement powders hydrolyzed under different complex enzyme addition amount were further characterized. Mix the raw material with water (1:6, *w*/*v*), add cellulase (0.50%), and perform enzymatic hydrolysis at 55 °C for 50 min followed by enzyme deactivation at 100 °C for 5 min. Subsequently, add composite enzyme (amylase: glucosidase = 1:3, *w*/*w*) at different ratios (0% (OC), 0.5% (CE_1), 1.0% (CE_2)), and conduct enzymatic hydrolysis at 50 °C for 40 min. After enzyme deactivation at 100 °C for 5 min, perform suction filtration and drying. The samples were then sieved (100 mesh) and stored.

The moisture content (GB 5009.3-2016) [15], protein content (GB 5009.5-2025) [16], ash content (GB 5009.4-2016) [17], and fat content (GB 5009.6-2016) [18] were measured using standard methods (Table S2).

### 2.3. Color Difference Analysis

Using a CR-10 Plus colorimeter (Konica Minolta, Tokyo, Japan), the colors L*, a*, and b* were measured. The L* value represents brightness and ranges from 0 (black) to 100 (white); the a* values represent positive and negative readings for red and green, respectively; the b* values represent positive and negative readings of yellowness and blueness, respectively [19]. In addition, the hue and chroma were determined using the following equations:

(1) $$\mathrm{Hue}(h*)=\tan{\left(b^{*}/a^{*}\right)}^{-1}$$

(2) $$\mathrm{Chroma}(c*)=\sqrt{{a*}^{2}+{b*}^{2}}$$

### 2.4. Determination of Dispersion, Wettability and Hydration Properties

The sample (1 g) was added to distilled water (25 mL) and magnetically stirred at a speed of 300 r/min until the sample was fully dissolved. The time required for complete dissolution of the sample was recorded.

The sample (1.0 g) was quickly and evenly distributed on the surface of the distilled water (50 mL, 50 °C), and the entire wetting time of the sample was recorded [20].

The determination of hydration properties was adapted from Heo et al. [21] with modifications. Specifically, dried sample (0.5 g) was homogenized with 20 mL distilled water. The mixture was subsequently subjected to thermal treatment in two separate water baths: first at 25 °C for 30 min followed by 100 °C for another 30 min, with intermittent stirring every 5 min. After thermal processing, the solution was centrifuged at 10,000 r/min for 30 min. The obtained supernatant was dried to constant weight in a forced-air oven at 105 °C. The soluble solid content was calculated using the following equation:

(3) $$\mathrm{Water}\;\mathrm{Absorption}\;\mathrm{Index}\;(\mathrm{WAI})=m_{1}/m$$

(4) $$\mathrm{Water}\;\mathrm{Solubility}\;\mathrm{Index}\;(\mathrm{WSI})=m_{2}/m\times 100\%$$

(5) $$\mathrm{Swelling}\;\mathrm{Power}\;(\mathrm{SP})=m_{1}/m\times (1-\mathrm{WSI}/100)$$

where: m is the dry weight of the sample/g; m_1_ is the weight of the sediment/g; m_2_ is the weight of the supernatant/g.

### 2.5. Determination of Water and Oil Holding Capacity

#### 2.5.1. Determination of Water-Holding Capacity (WHO)

Mix the appropriate amount of sample with deionized water, shake evenly and let stand at room temperature for 60 min, and then centrifuge for 15 min (rotation speed 3000 r/min).

(6) $$\mathrm{Water}\;\mathrm{holding}\mathrm{capacity}/\left(g/g\right)=\left(m_{1}-m_{2}\right)/m_{0}$$

where: m_0_ is the sample mass/g; m_1_ is the centrifuge tube mass/g; m_2_ is the sample and centrifuge tube mass/g after water absorption.

#### 2.5.2. Determination of Oil Holding Capacity (OHC)

According to Chau [22] method and slightly adjusted, the appropriate amount of sample and soybean oil were mixed in a centrifuge tube, and allowed to stand in a water bath at 37 °C for 4 h for 15 min (rotation speed 4000 r/min).

(7) $$\mathrm{Oil}\;\mathrm{holding}\mathrm{capacity}/\left(g/g\right)={(m}_{1}-m_{0})/m_{0}$$

where: m_0_ is the mass of the sample/g; m_1_ is the mass of the sediment in the centrifuge tube/g.

### 2.6. Determination of Antioxidant Capacities

The DPPH and ABTS scavenging activities were determined according to the method by Donoso-Bustamante, et al. [23].

### 2.7. Determination of Particle Size

The particle size distribution of the sample was measured by a laser particle size distribution meter (Bettersize2000, Dandong, China) at a pump speed of 1600 rpm. The refractive index of the sample was 1.52.

### 2.8. Fourier Transform-Infrared Spectroscopy (FT-IR)

The powder was ground and mixed with potassium bromide (KBr) at a ratio of 1:150 and pressed into tablets. The secondary structure content of the samples was analyzed by FTIR spectrometer (IRTracer-100, Kyoto, Japan) with 32 scans in the range of 400–4000 cm^−1^ [24].

### 2.9. Absorption Characteristics

#### 2.9.1. *In Vitro* Sodium Cholate Adsorption

The bile salt binding capacity was determined *in vitro* under simulated conditions of gastrointestinal tract, referring to the method reported by Chen et al. [25]. The samples were added into the artificial gastric juice (1:20, *w*/*v*) and oscillated in a 37 °C water bath for 1 h (100 r/min). The pH of the solution was adjusted to 6.3, then an equal volume of artificial intestinal fluid was added, and the solution was oscillated in a 37 °C water bath for 1 h (100 r/min). After digestion *in vitro*, 4 mL cholate solution was added to each sample, and the sample was oscillated in a water bath for 1 h (100 r/min, 37 °C). After centrifugation at 4000 r/min for 20 min, the content of cholate in the supernatant was measured. The calculation formula is as follows:

(8) $$Adsorption\;capacity\left(\frac{\mu mol}{100}mg\right)=(m_{1}-m_{2})/m_{0}$$

where: m_0_ is the mass of the sample; m_1_ is the mass of sodium cholate before adsorption; m_2_ is the mass of sodium cholate after adsorption.

#### 2.9.2. Cholesterol Adsorption Capacity (CAC)

Fresh egg yolks were diluted with 9 times the volume of distilled water. A total of 50 mL diluted egg yolk was added to the sample (2 g) and the pH was adjusted to 2.0 and 7.0, respectively. The solution was shaken at 37 °C for 2 h and centrifuged for 20 min (4000 rpm). The content of cholesterol in supernatant was determined by o-phthalaldehyde method [26], and the concentration of cholesterol was determined by standard curve equation (y = 1.1276x + 0.0607, R^2^ = 0.9908).

### 2.10. In Vitro Digestion Characterization

*In vitro* digestion characteristics were conducted according to Englyst et al. [27]. In short, 200 mg of sample was evenly mixed with 15 mL of sodium acetate buffer (pH = 5.2), then 5 mL preheated mixed enzyme solution (290 U/mL α-amylase, 15 U/mL glucoamylase) was added. Enzymatic hydrolysis was oscillated in a constant temperature water bath at 37 °C and 150 r/min, and samples were taken regularly. Glucose concentration was determined by measuring absorbance at 540 nm using the DNS method, with a standard curve established for carbohydrate digestion (CHO) calculation. The calculation formula is as follows:

(9) $$CHO(\%)=m_{1}*0.9/m_{2}*100$$

(10) $$RDS\left(\%\right)=\left[\left(G20-FG\right)/TS\right]\times 0.9\times 100$$

(11) $$SDS\left(\%\right)=\left[\left(G120-G20\right)/TS\right]\times 0.9\times 100$$

(12) $$RS\left(\%\right)=1-RDS-SDS-FG$$

where: m_1_ is the amount of glucose released/mg; m_2_ is the mass of sample (starch)/mg; 0.9 is the conversion factor, which can convert glucose into starch; G20 and G120 represent the glucose content released after 20 min and 120 min respectively; FG is the free glucose content; TS is the total starch content.

The hydrolysis index (HI) is the ratio of the area under the hydrolysis curve of starch (sample) to that of white bread (reference sample). Estimate the glycemic index (eGI) according to the formula [28]:

(13) HI=(AUC/rAUC) ×100

(14) eGI=0.862HI+8.1981

where: AUC is the area of the sample digestion curve; rAUC is the area of the reference sample digestion curve.

### 2.11. In Vitro Gastrointestinal Digestive Characterization

The sample was mixed with ultrapure water (10% *w*/*v*) and combined with 20 mL simulated gastric fluid at a 1:1 (*v*/*v*) ratio. The mixture pH was adjusted to 2.5 using 0.1 M HCl/NaOH and incubated in a shaking water bath (37 °C, 150 rpm) for 2 h. Aliquots were collected at 30 min intervals during incubation. Enzymatic reactions were terminated by boiling for 5 min, followed by centrifugation (8000× *g*, 10 min, 4 °C). Both supernatant and pellet fractions were collected for subsequent analysis.

The gastric digestate (30 mL) was neutralized to pH 6.8 ± 0.1 using 0.2 M NaOH, followed by addition of 1.5 mL preheated small intestinal fluid (SIF) and 3.5 mL bile salts with subsequent pH readjustment. After 2 h incubation at 37 °C (with 30 min interval sampling), enzymatic activity was terminated by boiling for 5 min. The samples were centrifuged (8000 rpm, 10 min) to separate supernatants and precipitates for preservation.

Samples were taken at different time periods of the gastrointestinal digestion process to determine the change in glucose release rate of different samples with hydrolysis time. The glucose concentration was determined using the 3,5-dinitrosalicylic acid (DNS) method to measure the absorbance at a wavelength of 520 nm [29].

### 2.12. Everted Intestinal Sac Model

Male Sprague–Dawley (SD) rats weighing 200 ± 20 g were obtained from Jinan Pengyue Experimental Animal Breeding Co., Ltd. (Jinan, China). All animals were adaptively fed for one week (23 ± 2 °C, 12 h light-dark cycle) before the experiment. The rats were randomly divided into 4 groups, with 3 rats in each group (n = 3). All animal procedures were approved by the Ethical Committee for the Experimental Use of Animals, Yantai University (YDLL2024R051). According to our previously established methodology [30], rat everted gut sac models were constructed to investigate glucose absorption characteristics of samples in different intestinal segments.

The intestinal segments were everted to position the serosal side inward, and both ends were ligated to custom-made cannulas. The prepared gut sacs were incubated in sample-containing Tyrode solution (37 °C) continuously oxygenated with carbogen (95% O_2_ + 5% CO_2_). Sampling was performed at predetermined time intervals (15, 30, 45, 60, 90, 120, and 180 min), with equivalent volumes of blank Tyrode solution replenished after each sampling to maintain constant fluid levels.

### 2.13. In Vitro Fecal Fermentation Characterization

#### 2.13.1. Sample Collection

Fecal samples were collected from six healthy adult volunteers (n = 6) recruited from the student population at Yantai University, and the study protocol was approved by the Ethics Committee of Yantai University (YDLL2024H010). Fresh fecal samples were collected within 3 h of excretion from six healthy volunteers (3 males and 3 females, age range 22–30 years). Inclusion criteria required participants to maintain normal body mass index (BMI) values (18.5 kg/m^2^ < BMI < 23.9 kg/m^2^) and meet the following health conditions: (1) absence of gastrointestinal disorders, (2) no reported use of tobacco products or alcohol consumption, (3) abstinence from probiotic-containing foods or supplements during the preceding week, and (4) no history of antibiotic treatment within the six months prior to sample collection. Those who met the requirements were selected through pure random sampling to ensure the randomness of the fecal samples. Each sample should be of equal weight and mixed evenly to ensure a balanced source of samples. Fecal samples were homogenized with anaerobically pre-reduced phosphate-buffered saline (PBS) in a 1:3 (*w*/*v*) ratio, followed by filtration through four layers of sterile cheesecloth to obtain a fecal slurry. The resulting filtrate was further diluted with fresh PBS at a 1:4 (*v*/*v*) ratio, and all aliquots were immediately transferred into anaerobic reaction tubes sealed to maintain strict anoxic conditions. Samples were subsequently incubated in a temperature-controlled anaerobic chamber (YQX-II, Xiqian, China) at 37 °C for 24 h to simulate colonic fermentation.

#### 2.13.2. Determination of Short-Chain Fatty Acids Content

Short-chain fatty acid (SCFA) quantification was performed using GC-2014 gas chromatograph (Shimadzu, Japan) equipped with a flame ionization detector and an Agilent (Santa Clara, CA, USA) Nukol™ capillary column (30 m × 0.53 mm × 0.5 μm) [31]. Analytical separation was achieved with helium carrier gas at 2.0 mL/min flow rate, using a programmed temperature gradient from 80 °C to 220 °C at 10 °C/min.

#### 2.13.3. 16S rDNA Sequencing Analysis

The total fecal microbial DNA was obtained through the Fecal Genome DNA Extraction Kit (AU46111-96, Beijing, China) according to the manufacturer’s instruction manual. Samples were further sequenced and analyzed by Shanghai Biotree Biotech Co., Ltd. (Shanghai, China). The DNA was quantified by Qubit (Invitrogen, Carlsbad, CA, USA). Qualifed PCR products were evaluated using an Agilent 2100 Bioanalyzer (Santa Clara, CA, USA) and Illumina library quantitative kits (Kapa Biosciences, Woburn, MA, USA), which were further pooled together and sequenced on an Illumina NovaSeq 6000 (PE250, Santa Clara, CA, USA).

### 2.14. Statistical Analysis

All experimental data were expressed as mean ± standard deviation. Statistical analyses were conducted using IBM SPSS Statistics (version 20.0, IBM Corp., Armonk, NY, USA). Significant differences among groups were determined by one-way analysis of variance (ANOVA) followed by Duncan’s multiple range test at a 95% confidence level (*p* < 0.05). Graphical representations were generated using OriginPro 9.1 (OriginLab Corporation, Northampton, MA, USA).

## 3. Result and Discussion

### 3.1. Effect of Enzymatic Hydrolysis Treatment on Color

Color serves as a key determinant in consumer food choices. Enzymatic hydrolysis significantly modified the chromatic properties (L*, a*, b*, c*, h*) of meal replacement powder (Table 1). The L* value (brightness indicator) decreased with treatment, indicating darkening. Both a* (red–green axis) and b* (yellow–blue axis) values showed dose-dependent reductions, particularly in yellowness (b*). Hue angle (h*), distinguishes color types, decreased by 49% (52.13° to 26.63°) when enzyme concentration doubled from 0.0% (OC) to 1.0% (CE_2), while chroma (c*) shifted from 7.57 to 5.32, reflecting diminished redness dominance. These alterations (*p* < 0.05 vs. untreated control group) demonstrate enzymatic treatment reduces color vibrancy and shifts hue toward muted tones, aligning with proanthocyanidin-rich starch systems [32]. Given color’s established role in food acceptance [33], this trade-off between functional enhancement (via enzymatic treatment) and sensory quality necessitates optimization. Further research should quantify how specific enzymatic modifications affect consumer preference thresholds.

### 3.2. Effect of Enzymatic Hydrolysis Treatment on Dispersion, Wettability and Hydration Characteristics

As quantitatively demonstrated in Table 2, controlled enzymatic hydrolysis induced dose-dependent enhancement of hydration properties in the meal replacement powder. Both CE_1 and CE_2 formulations displayed significant improvements in reconstitution efficiency compared to the OC control, manifesting as 25.70% and 34.61% acceleration in dispersion rates, coupled with 36.28% and 43.40% enhancement in wetting kinetics, respectively (*p* < 0.05). These functional improvements correlated with structural modifications including disrupted starch granule integrity, which enhanced water accessibility and molecular mobility [34]. Quantitative analyses revealed concomitant elevation of water absorption index (WAI) and water solubility index (WSI), while swelling power (SP) exhibited a 74.43% reduction at 100 °C (*p* < 0.05), indicative of fundamentally altered starch-water interaction patterns.

The observed enhancements stem from two synergistic structural mechanisms: (1) enzymatic depolymerization: targeted cleavage of α-1,4-glycosidic bonds by amylolytic enzymes converted high-molecular-weight starch polymers into low-molecular-weight dextrins and oligosaccharides. This structural modification achieved dual effects: exposure of latent hydrophilic groups through polymer chain scission, and disruption of crystalline domains through hydrogen bond network disassembly, ultimately generating porous matrices with enhanced capillary water transport capacity [35]. (2) Interfacial modulation: the hydrolysis-derived peptide-oligosaccharide complexes functioned as amphiphilic biosurfactants, effectively reducing interfacial tension between hydrophobic starch fragments and aqueous media. This surface-active property facilitated particle dispersion through electrostatic stabilization and steric hindrance effects [36].

### 3.3. Effect of Enzymatic Hydrolysis Treatment on Oil and Water Holding Capacity

The oil- and water-holding capacities (OHC/WHC) of meal replacement powders constitute critical functional indices, reflecting respective lipid and water retention capabilities post-centrifugation while providing insights into matrix molecular interactions [37]. As delineated in Table 2, enzymatic hydrolysis elicited differential responses in these parameters: OHC demonstrated concentration-dependent enhancement with 12.63% (CE_1) and 16.32% (CE_2) increases, whereas WHC exhibited progressive reductions of 6.61% and 17.23%, respectively, relative to the untreated control (OC).

Enzymatic hydrolysis enhances oil-holding capacity (OHC) via structural modification: macromolecular breakdown creates porous matrices with increased surface area (SSA) for oil entrapment, while exposing lipophilic groups (e.g., aliphatic chains) strengthens hydrophobic interactions. Conversely, water-holding capacity (WHC) declines as the fragmented structure weakens matrix cohesion and reduces hydrophilic moieties (hydroxyl/carboxyl groups), facilitating water displacement [38]. This functional dichotomy necessitates enzyme selection balancing lipid retention and water-binding loss. Future studies should correlate enzyme specificity with molecular architecture-hydration dynamics to optimize meal replacement functionality.

### 3.4. Effect of Enzymatic Hydrolysis Treatment on Cholate/Cholesterol Adsorption and Antioxidant Capacity

Enzymatic modification with compound enzymes substantially improved bile salt adsorption capacities of meal replacement formulations (Table 3). Compared to the OC control, CE_1 and CE_2 groups exhibited 9.70% and 64.15% enhancements in sodium taurocholate binding, respectively, with parallel increases of 40.27–54.52% in sodium glycocholate adsorption. Mechanistically, enzymatic hydrolysis induces structural reorganization of macromolecules, exposing hydrophobic domains that preferentially bind bile salts through enhanced surface interactions [39]. This modification strategy, however, displayed an inverse correlation with cholesterol adsorption efficiency, showing 15.63% (CE_1) and 18.75% (CE_2) reductions compared to controls. These modified formulations show potential for metabolic syndrome management through dual lipid regulation mechanisms [40].

Concurrently, enzymatic treatment enhanced antioxidant capacity, as shown in Table 3. Both ABTS^+^ and DPPH radical scavenging rates increased proportionally with enzyme concentration: 4% (0.5% dose) and 6% (1.0% dose) improvements for DPPH, with consistent 4% gains for ABTS^+^ across concentrations. These findings highlight the importance of enzymatic modification as a viable strategy to enhance the functional properties of meal replacement formulations.

### 3.5. Effect of Enzymatic Hydrolysis Treatment on In Vitro Digestion Characteristics

#### 3.5.1. *In Vitro* Digestion Characteristics

**Figure 1:**
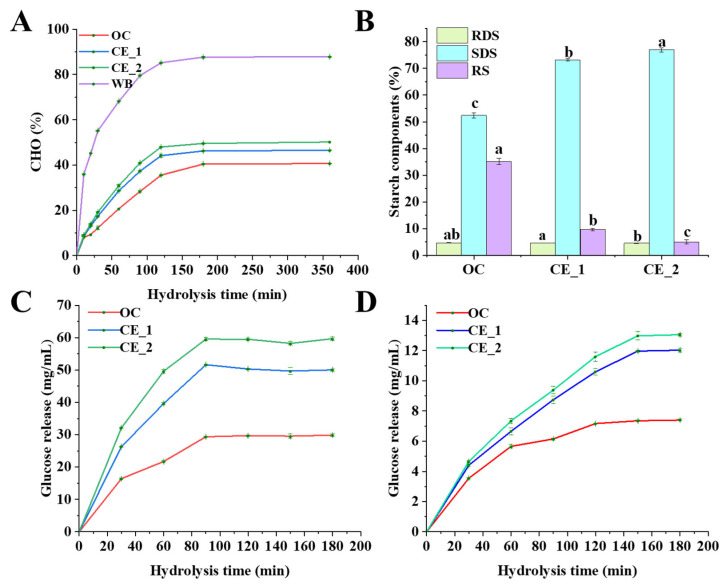
*In vitro* digestion characteristics. (**A**): The hydrolysis rate of carbohydrates (CHO); (**B**): starch fraction in different treatment groups (%); (**C**,**D**): glucose release during gastric (**C**) and intestinal (**D**) digestion. Different letters indicate statistical differences (*p* < 0.05). WB: white bread; RDS: rapidly digestible starch; SDS: slowly digestible starch; RS: resistant starch.

As shown in Figure 1A, the *in vitro* starch digestion kinetics of meal replacement formulations were systematically evaluated through time-dependent enzymatic hydrolysis monitoring. White bread (WB) demonstrated the most rapid starch hydrolysis, with CE_2 and CE_1 showing intermediate digestion rates, whereas OC sample displayed the lowest digestibility (Figure 1A). Figure 1B demonstrates significant compositional differences in starch fractions across samples. While no significant variation was observed in rapidly digestible starch (RDS) content among CE_1, CE_2 and OC (Figure 1B), striking contrasts emerged in their slowly digestible (SDS) and resistant starch (RS) components. Quantitative analysis showed CE_1 and CE_2 group contained 20.80% and 24.54% more SDS, respectively, compared to OC group. Conversely, OC group retained 25.53–30.17% higher RS levels than its enzyme-treated counterparts (*p* < 0.05), highlighting the inverse relationship between enzymatic modification and RS preservation. This digestion hierarchy correlates with structural modifications achieved through enzymatic pretreatment. Specifically, α-amylase treatment in CE_1 and CE_2 effectively disrupted starch granule integrity and crystalline domains, producing oligosaccharide fragments with enhanced enzymatic accessibility during simulated digestion. It is these changes that show that compared with the OC group of natural meal replacement powder, the digestive characteristics of meal replacement powder have been changed after enzymatic decomposition [41].

#### 3.5.2. Estimated Glycemic Index

The glycemic index (GI), a crucial nutritional metric defined by ISO 26642:2010 [42], quantifies the postprandial glycemic response magnitude and kinetics. As demonstrated in Table 3, both engineered formulations exhibited significant hypoglycemic advantages over conventional WB (94.40 ± 0.18), with CE_1 (47.93 ± 0.29) and CE_2 (51.40 ± 0.14) achieving certified low-GI status (<70). Notably, CE_2′s glycemic profile (51.40 ± 0.14) approaches the clinically significant <55 GI threshold recommended for diabetic management, positioning it as a potential transitional option for populations requiring intermediate glycemic regulation [43]. This enzymatic modification strategy achieved metabolic equivalence to historical low-GI meal replacements (e.g., quinoa-based system: 53 ± 4.0 GI [44] through advanced structural engineering rather than compositional reformulation—a paradigm shift offering distinct advantages in manufacturing scalability and ingredient flexibility compared to traditional substitution approaches.

#### 3.5.3. Simulated Gastrointestinal Digestion Characteristics

The time-dependent glucose liberation profiles during simulated gastrointestinal digestion (Figure 1C,D) exhibited inverse correlations with predicted glycemic responses, conforming to classic carbohydrate digestion dynamics [45]. Phase I digestion (0–100 min) displayed distinct formulation-specific release patterns: CE_2 > CE_1 > OC, with release kinetics transitioning to pseudo-steady state conditions during Phase II (100–180 min). This biphasic behavior likely reflects progressive depletion of accessible hydrolyzable bonds coupled with enzyme saturation kinetics [46]. In addition, during the intestinal digestion phase, although the enzyme treated group had more total glucose release, the time to peak was shorter. Together, these mechanistic insights validate the superior performance of different processed meal replacements in glycemic regulation through controlled nutrient release kinetics.

#### 3.5.4. Fourier Transform Infrared Spectroscopy (FT-IR)

**Figure 2:**
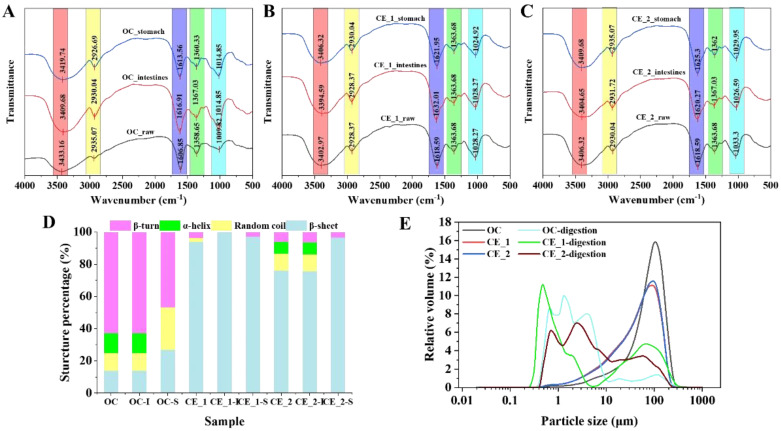
Fourier transform infrared spectroscopy and particle size of samples before and after digestion. (**A**–**C**): Fourier transform infrared spectroscopy; (**D**): secondary structure; (**E**): particle size.

FT-IR analysis systematically characterized the structural evolution of meal replacement powder during simulated gastrointestinal digestion. Comparative spectroscopic evaluation of the OC, CE_1, and CE_2 cohorts demonstrated absence of novel absorption bands while indicating nuanced variations in both peak architecture and relative absorbance intensities (Figure 2A–C). FT-IR exhibited characteristic vibrational modes: a broad hydroxyl (-OH) stretching vibration at 3400 cm^−1^, C-H stretching at 2930 cm^−1^, C=O stretching at 1620 cm^−1^, and C-O-C stretching vibrations between 1010–1030 cm^−1^. The absorption at 1360 cm^−1^ was attributed to combined C-N stretching and N-H bending vibrations [47].

The FT-IR spectra of all investigated samples displayed remarkably consistent characteristic absorption profiles (Figure 2), suggesting that diverse digestive conditions primarily induced structural reorganization through molecular chain realignment rather than covalent bond formation or cleavage. This phenomenon can be mechanistically interpreted through enzymatic dynamics in simulated gastrointestinal environments: (1) carbohydrate structural evolution: progressive attenuation of C-O-C glycosidic linkage signals (1000–1200 cm^−1^) corresponded to α-amylase-mediated polysaccharide depolymerization; blue shifts in C-H (1300–1500 cm^−1^) and C-O (1000–1200 cm^−1^) vibrational modes indicated molecular chain shortening effects; enhanced hydroxyl group interactions (3200–3600 cm^−1^) reflected increased monosaccharide exposure during starch hydrolysis [48]. (2) Protein structural modifications: intensity reduction in amide I (1600–1700 cm^−1^) and II (1500–1600 cm^−1^) bands demonstrated protease-induced peptide bond scission; spectral broadening and peak displacement suggested secondary structure transitions between α-helical and β-sheet conformations [49]. The observed spectral transformations originated from synergistic physicochemical interactions involving gastric acidification (pH modulation), enzymatic catalysis (pepsin/trypsin/amylase activity), and macromolecular structural reorganization. Crucially, the preservation of fundamental spectral signatures confirmed the absence of covalent bond transformations throughout the digestive process, emphasizing the physical nature of structural modifications.

#### 3.5.5. Particle Size Distribution

The particle size characteristics of meal replacement powders underwent significant alterations following different treatments (Figure 2E). Enzymatic pretreatment induced pronounced particle size diminution, with distribution profiles showing decreased modal diameters. Composite enzyme treatment reduced mean particle dimensions from 97.69 μm (original control, OC) to 68.33 μm (CE_1) and 69.99 μm (CE_2), accompanied by an approximate two-fold increase in specific surface area from 706.5 cm^2^/g to 1309 cm^2^/g (CE_1) and 1285 cm^2^/g (CE_2) (Table S3). This size reduction can be attributed to the hydrolysis-mediated depolymerization of starch and cellulose macromolecules into lower molecular weight constituents.

Notably, *in vitro* digestion profoundly altered particle size distribution patterns, resulting in a shift from unimodal to polymodal (bimodal/multimodal) configurations. The mean particle size of all samples decreased significantly post-digestion, indicating that the gastrointestinal phase broke down macromolecular material into smaller particles. Furthermore, the specific surface area of the OC group (1895 cm^2^/g) exceeded that of both CE_1 (679.2 cm^2^/g) and CE_2 (1118 cm^2^/g). This difference is likely attributable to the greater structural integrity of the OC samples, which were more resistant to digestive enzymes and less susceptible to rapid hydrolysis. Consequently, these samples maintained greater structural complexity and porosity after digestion, leading to an increased specific surface area [50]. These findings highlight the critical role of enzyme-mediated processing in designing powder systems with tailored digestibility and enhanced techno-functional properties.

### 3.6. Glucose Absorption Properties

**Figure 3:**
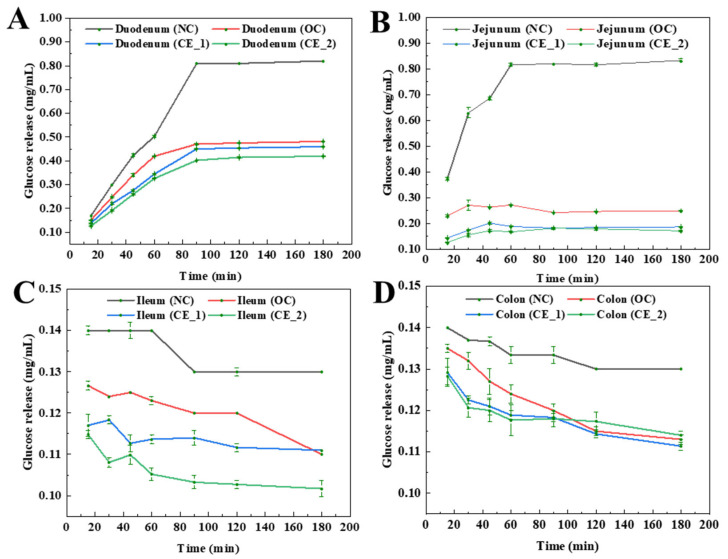
Glucose release and absorption properties of the sample in different intestinal segments. (**A**) Duodenum; (**B**) Jejunum; (**C**) Ileum; (**D**) Colon.

The glucose absorption profiles of samples across intestinal segments (duodenum, jejunum, ileum, and colon) at different timepoints are presented in Figure 3. In proximal intestinal segments (duodenum and jejunum), temporal analysis revealed distinct absorption patterns characterized by initial glucose accumulation followed by stabilization (Figure 3A,B). All modified formulations (OC, CE_1, CE_2) showed significant suppression of glucose release compared to NC, with jejunal segments demonstrating superior inhibitory capacity over duodenal counterparts. The enzymatic modification groups displayed dose-dependent efficacy, where CE_2 achieved maximal glucose suppression (62.3 ± 3.8% vs. NC), followed by CE_1 (54.1 ± 4.2%) and OC (46.9 ± 5.1%). In ileal and colonic segments, a progressive decline in glucose release was observed over time, with CE_1 and CE_2 groups demonstrating significantly lower release levels compared to NC and OC groups (*p* < 0.05). The hierarchy of glucose suppression efficacy followed the order: CE_2 < CE_1 < OC < NC, establishing a clear dose-response relationship between enzymatic hydrolysis intensity and glucose release reduction (Figure 3C,D). Notably, ileal segments exhibited more pronounced glucose-lowering effects than colonic segments across all treatment groups, suggesting enhanced inhibitory efficacy in the distal small intestine.

These findings demonstrate the segment-specific modulatory effects of enzymatically modified aronia meal replacements on intestinal glucose handling. The jejunum’s superior response to treatment versus duodenum may reflect regional differences in mucosal surface area and transporter density [51]. Notably, the sustained glucose-lowering capacity in colonic segments implies potential prebiotic effects mediated by microbial fermentation of resistant fractions [52]. From a nutritional perspective, the temporal-spatial modulation pattern supports the formulation’s potential for phased glucose regulation—rapid jejunal suppression followed by sustained ileal/colonic effects.

### 3.7. In Vitro Fermentation Characteristics

**Figure 4:**
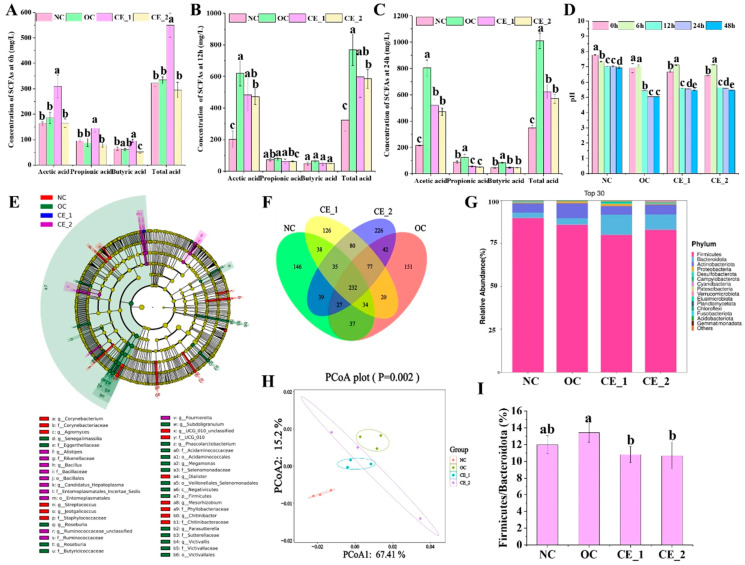
*In vitro* fermentation characteristics of different samples. (**A**–**C**): The content of short-chain fatty acids (SCFAs) during *in vitro* fecal fermentation; (**D**): pH value during *in vitro* fecal fermentation; (**E**): species evolution tree; (**F**): Venn diagrams; (**G**): the relative abundance at phylum level (24 h); (**H**): PCoA plot; (**I**): the ratio of *Bacteroidetes/Firmicutes*. Different letters indicate statistical differences (*p* < 0.05).

Increasing evidence highlights the critical role of short-chain fatty acids (SCFAs) in maintaining colonic energy homeostasis, with 60–70% of epithelial energy derived from these microbial metabolites [53]. As key microbial metabolites, short-chain fatty acids (SCFAs) play a crucial role in host metabolism and immunity through both local and systemic effects. Clinical trial results indicate that increasing the intake of indigestible but fermentable carbohydrates can ameliorate type 2 diabetes (T2D) phenotypes. This benefit is attributed to increased microbial SCFA production, with butyrate and propionate conferring specific metabolic benefits in glucose homeostasis and energy expenditure. Therefore, gut microbial fermentation of dietary fiber to produce SCFAs may confer metabolic benefits by modulating the gut microbiota [54]. *In vitro* fermentation results (Figure 4A–C) demonstrated progressive temporal increases in total SCFA concentrations for both native and enzymatically treated meal replacement formulations, indicating enhanced microbial biosynthetic capacity. Acetic acid predominated across all treatment groups, functioning dually as a primary energy substrate for enterocytes and hepatocytes and as a regulator of hepatic lipid and glucose metabolism [55]. Formulation-dependent metabolic divergence emerged, with CE samples exhibiting distinct SCFA profiles compared to OC groups, attributable to differential enzymatic accessibility of dietary fibers and slow-digesting starch complexes. Notably, the CE_1 group displayed unique phase-specific surges at 6 h in propionic acid (a liver-targeted hypocholesterolemic agent with cardioprotective effects) and butyric acid (the primary colonic energy source with anti-carcinogenic and barrier-strengthening properties) [56]. With increasing fermentation time (12 h and 24 h), acetic acid content increased significantly, while propionic and butyric acid levels decreased, suggesting interconversion between SCFAs. Consistent with the progressive accumulation of SCFAs, the pH of the samples gradually decreased over time (Figure 4D).

**Figure 5:**
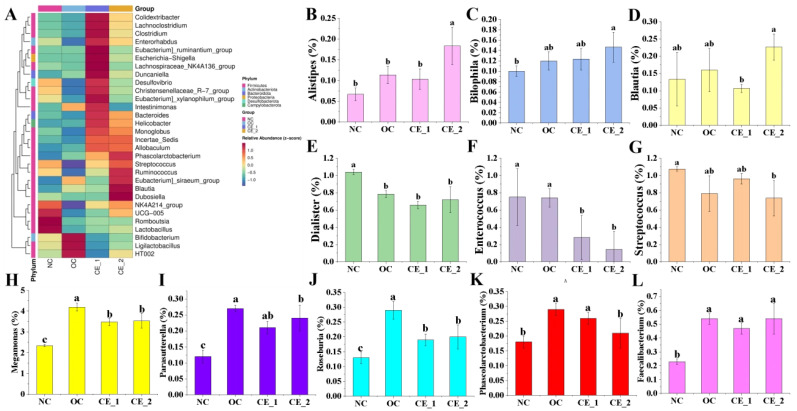
The relative abundance at genus level for 24 h. (**A**): Heatmap at genus level in fermentation; (**B**–**L**): relative abundance of *Alistipes*, *Bilophila*, *Blautia*, *Dialister*, *Enterococcus*, *Streptococcus*, *Megamonas*, *Parasutterella*, *Roseburia*, *Phascolarctobacterium*, and *Faecalibacterium*. Different letters indicate statistical differences (*p* < 0.05).

The species evolution tree of microbiota across different samples (Figure 4E) revealed significant structural reorganization of microbial communities among the intervention groups. This finding was corroborated by principal coordinates analysis (PCoA), which showed distinct β-diversity separation between the NC control and intervention groups (OC, CE_1, CE_2) along the PCoA1 (67.41% variance) and PCoA2 (15.2% variance) axes (Figure 4H). At the phylum level, the relative abundance of the top 30 microbial taxa is presented in Figure 4G. Firmicutes and Bacteroidetes dominated all groups, with significant variation observed between groups. Compared to the NC control, Bacteroidetes abundance increased 1.38-fold in the CE_1 group and 1.55-fold in the CE_2 group. However, the *Firmicutes/Bacteroidetes* (F/B) ratio (Figure 4I) did not change significantly following dietary intervention. Furthermore, analysis of genus-level flora characteristics (Figure 5B–L) demonstrated that, relative to the NC group, the intervention groups significantly promoted the growth of SCFA-producing bacteria (e.g., *Alistipes*, *Bilophila*, *Megamonas*, *Roseburia*, and *Blautia*) while inhibiting harmful bacteria (*Dialister and Enterococcus*) [57].

These findings establish enzymatic pretreatment as a strategic tool for the precision modulation of microbial metabolic routing. This enables the design of tailored SCFA production profiles: acetate-rich formulations for hepatic metabolic regulation versus butyrate-optimized systems for enhanced colonic health. Thus, enzymatic pretreatment bridges structural design with targeted physiological outcomes in functional food development.

## 4. Conclusions

Enzymatic hydrolysis significantly enhanced the physicochemical properties of the meal replacement powder, including color, hydration capacity, solubility, and antioxidant capacity, with these enhancements demonstrating dose dependency. *In vitro* gastrointestinal modeling demonstrated that the hydrolyzed powder exerted potent hypoglycemic effects, characterized by increased SDS content and delayed glucose release kinetics, resulting in lower GI values (CE_1: 47.93 ± 0.29; CE_2: 51.40 ± 0.14). Furthermore, enzymatic hydrolysis improved the bile salt adsorption capacity of the powder, effectively inhibited glucose release throughout the gut, and modulated intestinal flora composition and SCFA production. By elucidating the key role of enzymatic pretreatment in optimizing nutrient-microbiota interactions, this study advances precision nutrition and provides novel mechanistic insights for the dietary management of metabolic syndrome. Beyond the mechanistic insights, this study presents a foundation for the development of a functional food ingredient with tangible translational potential. The developed gut-targeted aronia meal replacement powder holds promise for clinical application, particularly as a dietary strategy for individuals concerned with glycemic management and gut microbiome homeostasis. Its design, leveraging enzymolysis for targeted colonic delivery, aims to maximize the localized bioactivity of aronia polyphenols and derived metabolites, potentially leading to improved clinical outcomes such as attenuated postprandial hyperglycemia and favorable shifts in gut microbial ecology as observed in our models. We emphasize that while our findings provide strong mechanistic support and proof-of-concept, well-controlled human clinical trials are essential to validate the observed benefits of glycemic control and microbial modulation, determine optimal dosing regimens, assess safety and tolerability in target populations, and evaluate the product’s efficacy within real-world dietary patterns. Future validation through randomized controlled trials in human populations focusing on endpoints like HbA1c, continuous glucose monitoring, specific microbial taxa abundance, and markers of gut barrier integrity will be essential to confirm its therapeutic utility as an adjunct to dietary guidelines.

## Figures and Tables

**Table 1:** Chromatic aberration analysis.

Sample	L*	a*	b*	c*	h*
OC	61.32 ± 0.73 ^a^	5.31 ± 0.03 ^a^	6.00 ± 0.14 ^a^	7.57 ± 0.14 ^a^	52.13 ± 0.72 ^a^
CE_1	55.13 ± 1.34 ^b^	4.66 ± 0.08 ^b^	3.02 ± 0.13 ^b^	6.10 ± 0.06 ^b^	29.53 ± 1.04 ^b^
CE_2	52.98 ± 0.89 ^c^	4.75 ± 0.20 ^b^	2.68 ± 0.17 ^c^	5.32 ± 0.32 ^c^	26.63 ± 2.45 ^b^

L*: brightness indicator, the range is from 0 (black) to 100 (white), used to measure the brightness of the color; a*: red–green axis, the positive value indicates a tendency towards redness, while the negative value indicates a tendency towards greenness; b*: yellow–blue axis, the positive value indicates a tint of yellow, while the negative value indicates a tint of blue; c*: chroma, the degree of vividness or saturation of a color; h*: hue angle, the types used to describe colors. Different letters indicate statistical differences (*p* < 0.05).

**Table 2:** Hydration characteristics, dispersibility, wettability, water-holding capacity, and oil-holding capacity of meal replacement powder.

Sample	Dispersibility (s)	Wettability (s)	WAI (g/g)		WSI (%)		SP (g/g)		OHC (g/g)	WHC (g/g)
			25 °C	100 °C	25 °C	100 °C	25 °C	100 °C		
OC	68.89 ± 1.21 ^c^	28.78 ± 1.28 ^b^	5.39 ± 0.34 ^b^	8.46 ± 0.61 ^b^	45.04 ± 1.39 ^b^	45.78 ± 1.29 ^b^	2.38 ± 0.18 ^a^	3.48 ± 0.37 ^a^	1.90 ± 0.08 ^b^	4.99 ± 0.44 ^a^
CE_1	51.18 ± 0.95 ^b^	18.34 ± 1.03 ^a^	6.57 ± 0.27 ^a^	10.00 ± 0.90 ^a^	64.14 ± 1.63 ^a^	76.06 ± 3.15 ^a^	1.43 ± 0.11 ^b^	1.14 ± 0.11 ^b^	2.14 ± 0.13 ^a^	4.66 ± 0.09 ^a^
CE_2	45.05 ± 0.62 ^a^	16.38 ± 1.16 ^a^	7.29 ± 0.32 ^a^	9.70 ± 0.67 ^ab^	65.26 ± 2.07 ^a^	80.04 ± 0.81 ^a^	1.43 ± 0.07 ^b^	0.89 ± 0.11 ^b^	2.21 ± 0.07 ^a^	4.13 ± 0.08 ^b^

WAI: water absorption index; WSI: water solubility index; SP: swelling power; WHC: water holding capacity; OHC: oil holding capacity. Different letters indicate statistical differences (*p* < 0.05).

**Table 3:** Effect of enzymatic hydrolysis on basic functional properties of meal replacement powder.

Sample	Sodium Taurocholate (μmol/100 mg)	Sodium Glycine Cholate (μmol/100 mg)	pH = 2 Cholesterol (mg/g)	pH = 7 Cholesterol (mg/g)	ABTS^+^ (%)	DPPH^+^ (%)	HI	eGI
OC	2.37 ± 0.06 ^c^	3.65 ± 0.09 ^c^	0.32 ± 0.01 ^a^	2.34 ± 0.04 ^a^	92.11 ± 1.91 ^b^	81.08 ± 0.94 ^c^	36.75 ± 0.25 ^c^	39.88 ± 0.22 ^c^
CE_1	2.60 ± 0.05 ^b^	5.12 ± 0.08 ^b^	0.27 ± 0.02 ^b^	1.60 ± 0.02 ^b^	96.34 ± 0.75 ^a^	85.40 ± 1.40 ^b^	46.09 ± 0.34 ^b^	47.93 ± 0.29 ^b^
CE_2	3.89 ± 0.05 ^a^	5.64 ± 0.12 ^a^	0.26 ± 0.01 ^b^	0.75 ± 0.02 ^c^	96.81 ± 0.43 ^a^	87.98 ± 0.66 ^a^	50.11 ± 0.16 ^a^	51.40 ± 0.14 ^a^

ABTS: 2,2′-Azino-bis (3-ethylbenzothiazoline-6-sulfonic acid) diammonium salt; DPPH: 2,2-diphenyl-1-picrylhydrazyl; HI: calculated hydrolysis indices; eGI: estimated glycemic index. Data (mean ± SD) in the same column with different letters indicate statistical differences (*p* < 0.05).

## Data Availability

The original contributions presented in the study are included in the article, and further inquiries can be directed to the corresponding authors.

## Supplementary Materials

The following supporting information can be downloaded at: https://www.mdpi.com/article/10.3390/foods14142456/s1, Table S1: Design and results of orthogonal experiment; Table S2: Nutrient Composition Determination Results; Table S3: Particle size analysis.
