# Supplementary material for: Enzymolysis-Driven Development of a Gut-Targeted Aronia melanocarpa Meal Replacement Powder with Glycemic Control and Microbial Homeostasis Benefits
Source: Foods. 2025 Jul 12;14(14):2456. doi: 10.3390/foods14142456 (PMC12296082; doi:10.3390/foods14142456)
Supplement: Supplementary file 1 [file foods-14-02456-s001.zip › Table S1.pdf]

Table S1 Design and results of orthogonal experiment

| | Factor | | | | DE value |
|---|---|---|---|---|---|
| | A Compound enzyme addition amount（%） | B Compound enzyme ratio （v/v） | C Time (min) | D Temperature (°C) | |
| 1 | 1(0.75) | 1(1:1) | 1(30) | 1(50) | 40.12±0.35 |
| 2 | 1(0.75) | 2(1:2) | 2(40) | 2(55) | 38.21±0.45 |
| 3 | 1(0.75) | 3(1:3) | 3(50) | 3(60) | 40.64±0.30 |
| 4 | 2(1.00) | 1(1:1) | 2(40) | 3(60) | 43.21±0.28 |
| 5 | 2(1.00) | 2(1:2) | 3(50) | 1(50) | 40.89±0.47 |
| 6 | 2(1.00) | 3(1:3) | 1(30) | 2(55) | 43.33±0.39 |
| 7 | 3(1.25) | 1(1:1) | 3(50) | 2(55) | 40.38±0.43 |
| 8 | 3(1.25) | 2(1:2) | 1(30) | 3(60) | 42.63±0.33 |
| 9 | 3(1.25) | 3(1:3) | 2(40) | 1(50) | 44.00±0.29 |
| K1j | 39.66 | 41.24 | 42.03 | 41.67 | |
| K2j | 42.48 | 40.58 | 40.64 | 40.64 | |
| K3j | 42.34 | 42.66 | 40.64 | 42.16 | |
| ′K1j | 13.22 | 13.75 | 14.01 | 13.89 | |
| ′K2j | 14.16 | 13.53 | 13.55 | 13.55 | |
| ′K3j | 14.11 | 14.22 | 13.55 | 14.05 | |
| R | 0.94 | 0.69 | 0.46 | 0.50 | |
| Priorities | A＞B＞D＞C | | | | |
| Best collection | $A_3B_3C_2D_1$ | | | | |
