# Supplementary material for: Enzymolysis-Driven Development of a Gut-Targeted Aronia melanocarpa Meal Replacement Powder with Glycemic Control and Microbial Homeostasis Benefits
Source: Foods. 2025 Jul 12;14(14):2456. doi: 10.3390/foods14142456 (PMC12296082; doi:10.3390/foods14142456)
Supplement: Supplementary file 1 [file foods-14-02456-s001.zip › Table S2.pdf]

Table S2 Nutrient Composition Determination Results

	Moisture (g/100g)	Protein (g/100g)	Fat (g/100g)	Dietary fiber (g/100g)	Ash (g/100g)	Starch (g/100g)
OC	10.43±0.01^{a}	16.49±0.11^{c}	1.23±0.15^{b}	29.37±0.42^{c}	1.75±0.12^{b}	50.05±0.99^{a}
CE_1	7.23±0.19^{b}	33.95±0.17^{b}	1.77±0.15^{a}	54.63±0.31^{b}	1.74±0.21^{b}	43.16±0.17^{b}
CE_2	7.41±0.20^{b}	35.24±0.38^{a}	1.80±0.10^{a}	56.73±0.87^{a}	2.09±0.12^{a}	40.55±0.41^{c}

Different letters indicate statistical differences ($p < 0.05$).
