# Supplementary material for: Enzymolysis-Driven Development of a Gut-Targeted Aronia melanocarpa Meal Replacement Powder with Glycemic Control and Microbial Homeostasis Benefits
Source: Foods. 2025 Jul 12;14(14):2456. doi: 10.3390/foods14142456 (PMC12296082; doi:10.3390/foods14142456)
Supplement: Supplementary file 1 [file foods-14-02456-s001.zip › Table S3.pdf]

Table S3 Particle size analysis

	Median diameter (D50) (μm)	Volume mean diameter [4,3] (μm)	Specific surface area (SSA) (cm^2/g)
OC	93.05	97.69	706.5
CE_1	57.71	68.33	1309
CE_2	60.05	69.99	1285
OC-digestion	2.043	116.0	1895
CE_1-digestion	1.806	120.3	679.2
CE_2-digestion	4.028	78.15	1118
